# Percolation-based precursors of transitions in extended systems

**DOI:** 10.1038/srep29552

**Published:** 2016-07-14

**Authors:** Víctor Rodríguez-Méndez, Víctor M. Eguíluz M, Emilio Hernández-García, José J. Ramasco

**Affiliations:** 1 Instituto de Física Interdisciplinar y Sistemas Complejos IFISC (CSIC-UIB), Campus Universitat de les Illes Balears, E-07122 Palma de Mallorca, Spain

## Abstract

Abrupt transitions are ubiquitous in the dynamics of complex systems. Finding precursors, i.e. early indicators of their arrival, is fundamental in many areas of science ranging from electrical engineering to climate. However, obtaining warnings of an approaching transition well in advance remains an elusive task. Here we show that a functional network, constructed from spatial correlations of the system’s time series, experiences a percolation transition way before the actual system reaches a bifurcation point due to the collective phenomena leading to the global change. Concepts from percolation theory are then used to introduce early warning precursors that anticipate the system’s tipping point. We illustrate the generality and versatility of our percolation-based framework with model systems experiencing different types of bifurcations and with Sea Surface Temperature time series associated to El Niño phenomenon.

The occurrence of sharp transitions to different states or regimes during the evolution of complex systems is a phenomenon of major importance both from the fundamental point of view and for practical implementations of control and management. Examples of such abrupt changes can be found in ecology^1^, economy^2^, electrical engineering^3^, physiology^4^ or climate^5^,^6^,^7^. Detecting with sufficient anticipation the approach to a critical or tipping point thus becomes an important issue. Early-warning signals have been introduced and tested in recent works^8^,^9^,^10^, including experimental verification in living and environmental systems^9^,^11^,^12^. These methods rely on the loss of resilience occurring generically when dynamical systems approach most (although not all) types of bifurcation points^6^,^10^,^13^. Recovery rates from perturbations become small, leading to *critical slowing down* of the dynamics, increased memory, long temporal autocorrelations, and to the growth of the temporal variance^6^,^10^,^13^. From a dynamical viewpoint, these critical slowing down phenomena appear when the eigenvalue of the Jacobian matrix describing the rate of relaxation towards the attractor approaches zero close to bifurcation points. In many cases, particularly when different spatial parts of the system are coupled by diffusion-like processes, the increase in temporal correlation is accompanied by the growth of spatial correlations^14^,^15^,^16^. This, with the associated increase in spatial variance and response functions, is actually a standard method to characterize phase transitions in thermodynamic physical systems^17^.

The consideration of spatial correlations has led to a novel perspective for finding transition precursors through the use of correlation or functional networks^18^,^19^,^20^,^21^. These networks are built by identifying spatial units as nodes in a graph, measuring the correlation among all pairs of them, and keeping the most significant ones as link weights. Several network-based precursors have been proposed. Specifically the values of the degree (number of connections per node), assortativity (degree-degree correlations), clustering (average density of triangles) and kurtosis rise when approaching a tipping point^22^,^23^,^24^.

Here we show an important additional property of these functional networks: As internal correlations increase, networks evolve from a low to a high connectivity state and, *before* reaching its maximum link density at the bifurcation point, a percolation-like transition occurs in the network topology. Concepts from graph percolation theory^25^,^26^ can thus be imported to characterize this transition. Importantly, metrics can be defined that act as early warnings for this percolation transition, which itself is a precursor of the dynamic transition. The validity of these general ideas is tested by analyzing model systems displaying different types of bifurcations: steady and oscillatory, continuous and discontinuous. In all cases, we observe the occurrence of percolation transitions before the dynamical one, and we characterize it with quantities that can be used as early-warning signals. Our approach uses only time series from the elements of an extended system, without the need of specific knowledge about the underlying dynamics. Thus, it is suited to analyze observational data for which little or no modeling insight is available. This property of our framework is illustrated by applying it to temperature data from the Pacific Ocean associated to El Niño phenomenon.

## Methods

### Functional networks and precursors

The time evolution of extended dynamical systems is described by time-dependent spatial fields. Let *ψ*(**x**, *t*) be one of such fields, and consider a suitable discretization of it {*ψ*(**x**_*l*_, *t*_*k*_)}_*l*,*k*_, defining a time-series at discrete times *t*_*k*_, *k* = 1, …, *R*, from each spatial location **x**_*l*_, *l* = 1, …, *N*. The construction of functional networks implies to compute the Pearson correlation from the time series at every pair of locations:


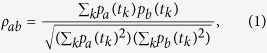


where 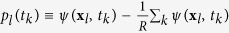 is the deviation of the field from its temporal mean at each location. A network in which nodes are the spatial locations **x**_*l*_ is defined by assigning links between pairs of nodes (**x**_*a*_, **x**_*b*_) for which the Pearson correlation in equation (1) is higher than a predefined threshold *γ: ρ*_*ab*_ > *γ*.

To study percolation in these functional networks, as the control parameter *p* approaches transition points *p*_*d*_, we measure several metrics: (a) *S*_1_, the relative size of the largest connected component, i.e. the fraction of nodes that are in the largest cluster. It abruptly changes from a value close to 0 to a value close to 1 at the percolation point. (b) The average size of the clusters excluding the largest one. This quantity is maximal at the percolation point^25^. It can be calculated as


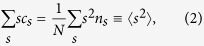


where the sum runs over all cluster sizes *s* excluding the largest one, and *c*_*s*_ is the fraction of nodes belonging to clusters of size *s, c*_*s*_ = *sn*_*s*_*/N (n*_*s*_ is the number of clusters of size *s* present in the system^26^) thus *c*_*s*_ also gives the probability that a randomly chosen node pertains to a cluster of size *s*.

The standard percolation indicators, *S*_1_ and 〈*s*^2^〉 are not, however, the best precursors. We show below that better anticipation can be obtained by exploiting the interplay between the probabilities *c*_*s*_ and the coming transition. In random graphs it is possible to perform analytic calculations on the behavior of *c*_*s*_. As new links are added at random, the percolation occurs when the mean degree 〈*k*〉, which acts as the control parameter *p*, equals one. Before this, the probabilities *c*_*s*_ that a randomly chosen node belongs to a component of size *s* can be written as


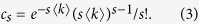


These probabilities have a maximum when the mean degree, which in this case is the parameter *p*, is 
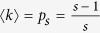
. The succession {*p*_*s*_} of the location of the maximum of *c*_*s*_ converges to the percolation point *p*_∞_ = 1 for increasing component size *s*, but for low *s* these maxima could be quite far from the percolation point, and in fact they anticipate it. We show below that the early-warning character of the maxima of *c*_*s*_ holds also true in non-random functional networks obtained from systems undergoing very different dynamic transitions. This is due to the generic increase in the dynamical correlations of the elementary units of the system that the approach of a global tipping point brings. The sequence of peaks in *c*_*s*_ offers thus a general and versatile tool to predict potential changes in the dynamics at a global scale.

### Percolation and transitions in model systems

We analyze here three different extended dynamical systems displaying different types of bifurcations. The first two examples experience steady bifurcations (a discontinuous saddle-node and a continuous pitchfork). The third case, the Lorenz’96 system, experiences a variety of transitions being the first one an oscillatory Hopf bifurcation between a steady state and traveling waves. Further transitions occur when changing the control parameter leading to low-coherence spatio-temporal chaos. In all cases we add random noise to the deterministic model. This represents the unavoidable stochastic fluctuations to which real systems are always subjected and provides the necessary statistics to have well-defined spatial correlation functions. In all cases percolation occurs in the associated functional networks, providing robust early-warning signals of the approaching transition.

## Results

### A lake eutrophication model

As a first example, we consider a lake eutrophication (LE) model which is a spatial version of a description of phosphorous recycling in a lake^27^. It suffers a paradigmatic abrupt transition associated to a saddle-node (SN) bifurcation, namely a transition between two contrasted states for the phosphorus concentration *ψ*(**x**, *t*) in the lake: low concentrations leading to clear water, and excess of phosphates leading to turbid water. The state of the system is given by the two-dimensional field *ψ*(**x**, *t*), representing the amount of phosphorus in the lake, evolving according to


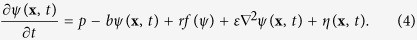


**Figure 1:**
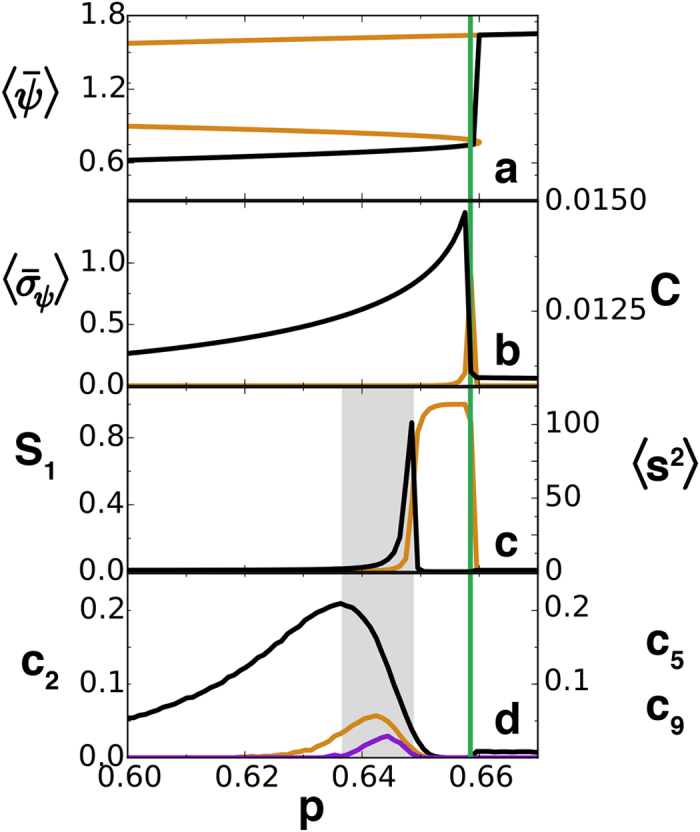
Transition precursors for the LE model, equation (4). In (**a**) the steady homogeneous phosphorus concentration *ψ* (orange) and the numerically obtained spatial average (black, further averaged over *R* = 1000 temporal snapshots for each value of *p*) as a function of the control parameter *p* which is slowly increased from low to high values. In (**b**) 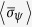, the spatial standard deviation (black) of *ψ* used in refs 13, 14, 15 as a transition precursor is displayed averaged over *R* = 1000 temporal snapshots. In orange, the clustering of the functional network built with threshold *γ* = 0.21 also used as a precursor in refs 22,23. In (**c**) the relative size of the giant component, *S*_1_ (orange), and the average size of the leftover clusters (〈*s*^2^〉, black). (**d**) The probabilities *c*_2_ (black), *c*_5_ (orange), and *c*_9_ (purple) are shown. In all the panels, the vertical green line marks the position of the observed abrupt transition (*P*_*d*_ = 0.658). The grey area indicates the anticipation in parameter space gained over previous methods by using the peak of *c*_2_ as precursor of the percolation transition. All curves have been further averaged over 100 realizations of the random noise and initial conditions.

The function 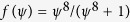 is a nonlinear response of the lake sediments to phosphorus, *p* is the nutrient input rate, taken here as the control parameter. *ε* is the strength of diffusive spatial coupling, and *η* represents an additive stochastic perturbation uncorrelated in space and time. We take *b* = *r* = 1 and *ε* = 1.2. Space is discretized as a square lattice of *N* = 70 × 70 = 4900 grid points separated by *dx* = 1. These will be the nodes of the functional network. The Laplacian is discretized with the simplest finite differences scheme and the deterministic terms in equation (4) are integrated with a 4th order Runge-Kutta method of time step *dt* = 0.05 after which the *η* term is implemented by adding an independent random number uniform in [−*a, a*] to each lattice site (we use *a* = 0.125). We approach from the left the SN bifurcation occurring at *p* = *p*_*SN*_ = 0.660, above which the clear-water low-phosphorus state existing for *p* < *p*_*SN*_ ceases to exist and the lake jumps to a eutrophicated high-phosphorus state. The stochastic perturbation makes the jump to occur at a value of *p, p*_*d*_, slightly below the SN value. On average (see Fig. 1), we find *p*_*d*_ ≈ 0.658. This value is sufficiently close to *p*_*SN*_ as to display the enhancement of correlations and slowing down which are at the basis of our method and of other early-warning methodologies. In this paper we show only results obtained when slowly increasing the control parameter *p*. When decreasing *p* from higher values hysteresis occurs and a different SN bifurcation is encountered at a lower 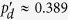. The sequence of precursors encountered when approaching this lower transition point is similar to the one shown here.

The increase of the spatial variance was used in refs 14,15 as transition precursor. This variance is defined for the discretized field *ψ*(**x**_*l*_, *t*_*k*_) in the asymptotic statistically steady state as:


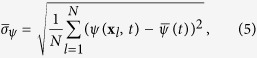


where 
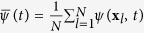
 is the average over the nodes. When the system approaches *p*_*d*_, the raise of spatial variance can be observed for the LE model (Fig. 1b).

As explained above, we constructed functional networks by assigning links between locations among which correlations (as measured by Pearson correlation) are larger than a threshold for which we use *γ* = 0.21. We take averages over *R* = 1000 temporal snapshots. The spatial correlations, computed from equation (1), increase and lead to a growth of the link density in the vicinity of the critical point as indicated by the precursors proposed in refs 22, 23, 24. In fact one of these network precursors, the clustering coefficient, is plotted in Fig. 1b (orange) and has a peak at the dynamical transition. But we will show that there is also a percolation transition, and to capture it we studied how the size of the giant component, *S*_1_, and the average size of the leftover clusters 〈*s*^2^〉 change with *p* (see Fig. 1c). Note that 〈*s*^2^〉 has a peak at *p* = 0.648, which identifies the occurrence of percolation in the network, way before the SN transition has happened, and thus it may be used as a signal that a dynamical transition is coming. The distance in the parameter space between that signal and the transition depends on which threshold is used to build the network, but there is an optimal value (see below).

The probabilities *c*_2_, *c*_5_ and *c*_9_ for the networks built from the LE correlations are shown in Fig. 1d. Their maxima clearly anticipate the percolation transition signaled by *S*_1_ and 〈*s*^2^〉, which gives itself an early warning of the SN bifurcation. The peak in *c*_2_ appears at *p*_2_ = 0.635, anticipating *p*_*d*_ more than twice as early as the percolation transition.

**Figure 2:**
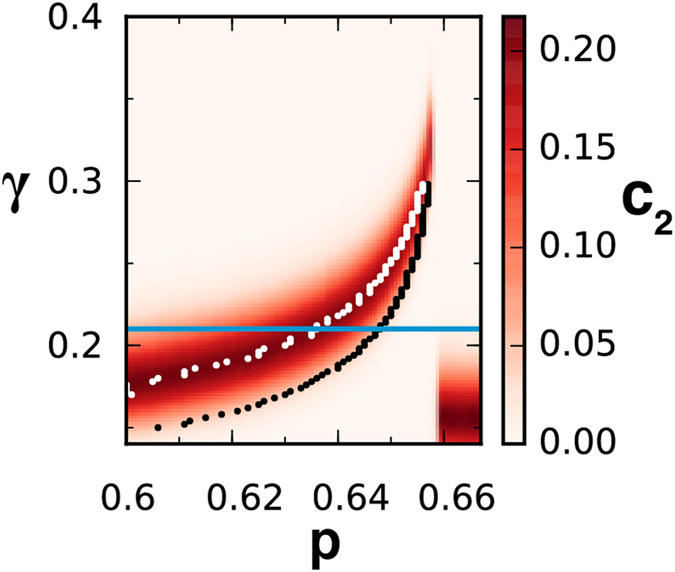
Role of the correlation threshold *γ*. *c*_2_ values, as given by the color bar, as a function of the control parameter *p* and the threshold *γ* used to build the functional network for the LE model. The white dots give the locations of the *c*_2_ maxima, while the black dots mark the maxima of 〈*s*^2^〉 (percolation transition). The dynamical sudden jump occurs at *p*_*d*_ = 0.658. The horizontal blue line identifies the value of *γ* = 0.21 used in Fig. 1.

Building functional networks requires setting the correlation threshold *γ* above which two elements are considered as linked. Figure 2 shows how the percolation transition and the values of *c*_2_ depend on both *p* and *γ*. If *γ* is very high, the network is never connected and there is no signal. Similarly, if *γ* is very low the network is always fully connected and there is no hint of the bifurcation. However, as shown in the figure, there is a range of values of *γ* where the percolation transition and its associated early warning signals appear. For a fixed value of *γ* the peak of *c*_2_ (white dots) always occurs earlier than the percolation transition (black dots). As *γ* decreases, the peak of *c*_2_ occurs at earlier values of *p*. The curve of *c*_2_, however, widens and the resolution in the location of the peak gets poorer. The lowest (optimal) value of *γ* at which the peak can be distinguished marks thus the earliest warning signal that can be obtained for the bifurcation. Note that this does not imply that the method only works for a fixed value of *γ*. In an empirical situation, one may need to explore this parameter, but there is a range of values of *γ* over the optimal that will provide valid early warning signals for the transition.

### The Ginzburg-Landau equation

To prove the generality of the precursors, we analyze a different system, the time-dependent Ginzburg-Landau (GL) equation or model A^28^ that describes, for example, transitions in anisotropic ferromagnets. It is a paradigmatic model experiencing a continuous transition, namely a supercritical pitchfork bifurcation. We study the one-dimensional version for the magnetization *ψ*(*x, t*):


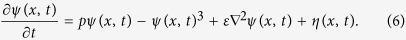


**Figure 3:**
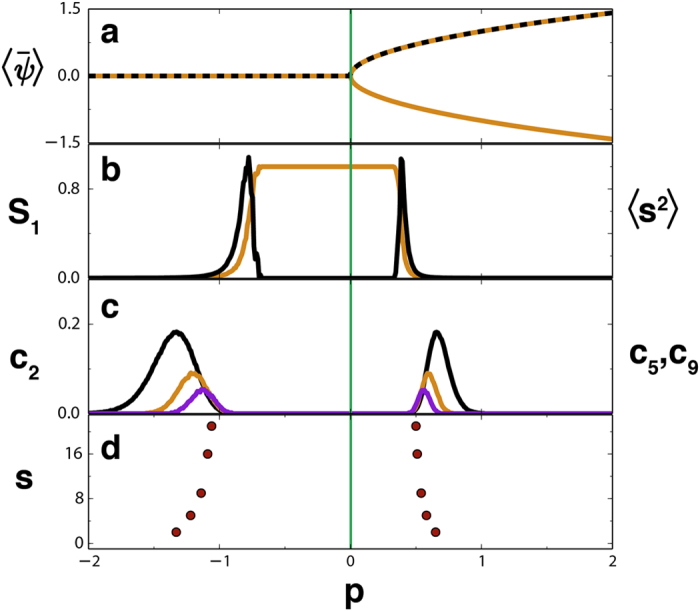
The Ginzburg-Landau model has a continuous transition at the critical value *p*_*d*_ = 0 marked by the green line. (**a**) The theoretical (yellow) and the numerically obtained (black dash line, further averaged over *R* = 1000 temporal snapshots) homogeneous value of the field *ψ*. (**b**) *S*_1_ (orange) and 〈*s*^2^〉 (black). (**c**) The quantities *c*_2_ (black), *c*_5_ (orange), and *c*_9_ (purple). (**d**) Circles indicate the values of *p, p*_*s*_ with *s* = 2, 5, 9, 16 and 21, for which *c*_*s*_ attains its respective maximum. Functional networks were built using a threshold of *γ* = 0.25.

As before, *ε* is diffusive coupling and *η* is an additive uncorrelated noise uniform in [−*a, a*]. We discretize equation (6) into *N* = 5000 nodes, and take *ε* = 1.5, *a* = 0.01. The integration parameters *dt* and *dx* are as for the LE model. A continuous transition from zero magnetization occurs when increasing the control parameter *p* above *p*_*d*_ = 0 (Fig. 3a).

We compute spatial correlations and build functional networks from *R* = 1000 snapshots using a threshold *γ* = 0.25. The corresponding percolation quantifiers are plotted in Fig. 3. Here correlations continuously build-up when increasing *p* towards the critical point at *p*_*d*_ = 0 and, unlike the previous discontinuous transition, continuously decrease after crossing it. Therefore, there are now two percolation transitions in the functional networks: one at each side of *p*_*d*_, as seen by the indicators *S*_1_ and 〈*s*^2^〉 in Fig. 3b. Figure 3c depicts the probabilities *c*_2_, *c*_5_ and *c*_9_, with maxima giving a clear warning further away from *p*_*d*_. Figure 3d displays the values *p*_*s*_ corresponding to these maxima in *c*_*s*_. There are two successions of peaks converging to the percolation transitions occurring before and after the bifurcation.

**Figure 4:**
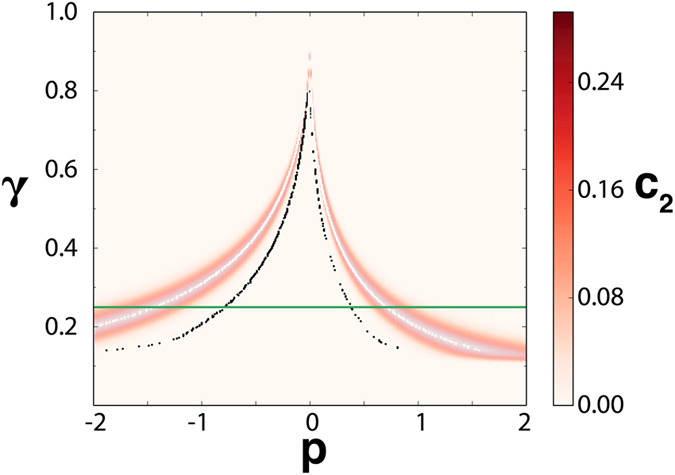
The *c*_2_ values, as given by the color bar as a function of the control parameter *p* and the threshold *γ* used to build the functional network for the Ginzburg-Landau system. The continuous pitchfork bifurcation occurs at *p*_*d*_ = 0. Black dots indicate the maxima of 〈*s*^2^〉, which locate the percolation transition. White dots locate the maxima of *c*_2_. We see how the percolation transition and its precursor *c*_2_ anticipate in different amounts (when increasing *p* from the low *p* state, or when decreasing *p* from the high *p* state) the dynamical bifurcation. The horizontal green line identifies the value *γ* = 0.25 for which Fig. 3 of the main text was constructed.

Figure 4 shows what happens to the percolation transition when building the networks for different thresholds *γ*. The figure displays the maxima of 〈*s*^2^〉 (which locate the percolation transitions) and the values of *c*_2_, in the (*γ, p*) parameter space. We see that, when increasing *p*, the maximum in the precursor *c*_2_ anticipates the percolation transition, which itself anticipates the pitchfork bifurcation at *p* = 0. At the other side of the transition, when decreasing *p* from the high *p* state, the maximum in *c*_2_ also occurs before the percolation transition, which also anticipates the dynamical transition. As in the LE case the amount of anticipation is larger for lower *γ*, until the signal disappears.

### The Lorenz’96 system

Coupled chaotic oscillators display a large variety of dynamical regimes. Thus, due to the different bifurcations present in those models, they are an excellent test bed to prove the generality of the network-based percolation precursors. Here we consider the Lorenz’96 model^29^. It was proposed by E. Lorenz as a simplified framework to investigate atmospheric predictability. It reads:


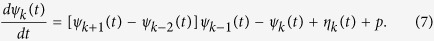


*ψ*_*k*_(*t*) is meant to represent the values of some atmospheric variable at different locations *k*, 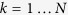, arranged in a one dimensional ring around the globe (and thus having periodic boundary conditions). The structure of equation (7) contains some of the main elements of fluid dynamics, namely dissipation, external forcing, and quadratic non-linearity through an advection-like term. In addition to the constant forcing *p*, which will be our control parameter, we include an additive stochastic perturbation *η*_*k*_(*t*) uncorrelated in space and time. It is implemented here by adding independent random numbers uniform in [−*a, a*] (*a* = 0.1) after each time step (*dt* = 1/64) of a fourth-order Runge-Kutta method which is used to integrate the rest of the terms. We focus in the behavior for *N* = 2500 elements or oscillators. In the absence of the random forcing, three dynamical regimes are easily identified^30^: (i) if *p* < *p*_1_ = 8/9, the system stabilizes in the homogeneous fixed point 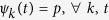; (ii) a Hopf bifurcation occurs at *p* = *p*_1_ so that for intermediate values of *p*, 8/9 < *p* < 4.1, the system is in a traveling-wave state; (iii) for *p* > *p*_2_ ≈ 4.1, the system becomes spatiotemporally chaotic.

**Figure 5:**
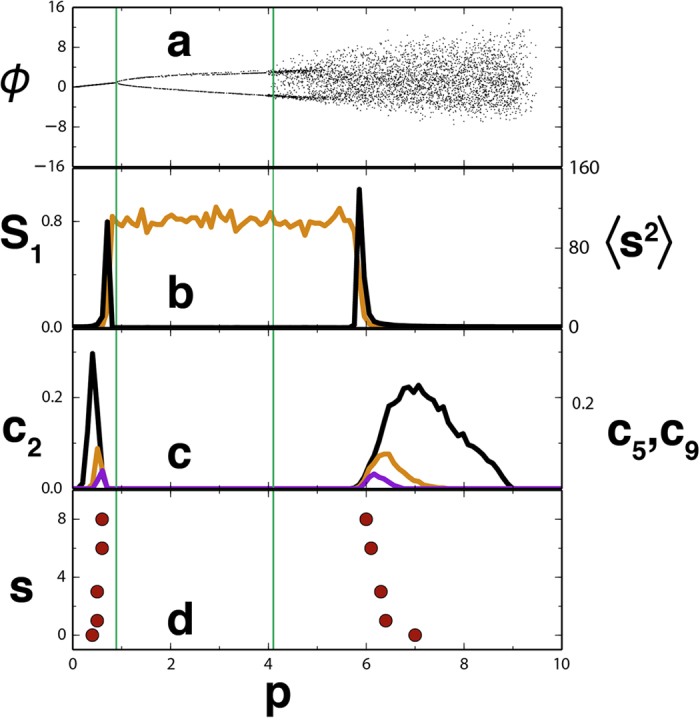
(Panel a) shows the bifurcation diagram of the Lorenz’96 model, equation (7), constructed from a 2-oscillator Poincaré section (see text). The transition to traveling waves at *p*_1_ = 8/9 and to spatiotemporal chaos (*p*_2_ ≈ 4.1) are shown as vertical green lines. Functional networks were built using a threshold of *γ* = 0.16. The percolation indicators *S*_1_ (orange) and 〈*s*^2^〉 (black) are displayed in (panel b). (Panel c) shows *c*_2_ (black), *c*_5_ (orange), and *c*_9_ (purple). In (**d**), the circles indicate the value of *p, p*_*s*_ with *s* = 2, 5, 9, 16 and 21, for which the *c*_*s*_ curves attain their respective maxima. These indicators reveal a phase of percolated correlations in a parameter region which includes the interval [*p*_1_, *p*_2_], flanked by two percolation transitions. The curves have been further averaged over 100 realizations of the random noise and initial condition.

To display the bifurcations observed when integrating equation (7) for *N* = 2500 elements we have calculated a Poincare’s transversal section in the subspace of two contiguous oscillators: one of the oscillators, say *ψ*_1_, is monitored and when it crosses the value *ψ*_1_ = 1 in the increasing direction the value of the contiguous oscillator, say *ϕ* = *ψ*_2_ is recorded and displayed. The bifurcation diagram showing the values of these sections *ϕ* is plotted in Fig. 5a as a function of *p*. The three regimes described above for the deterministic system are readily identified here also.

**Figure 6:**
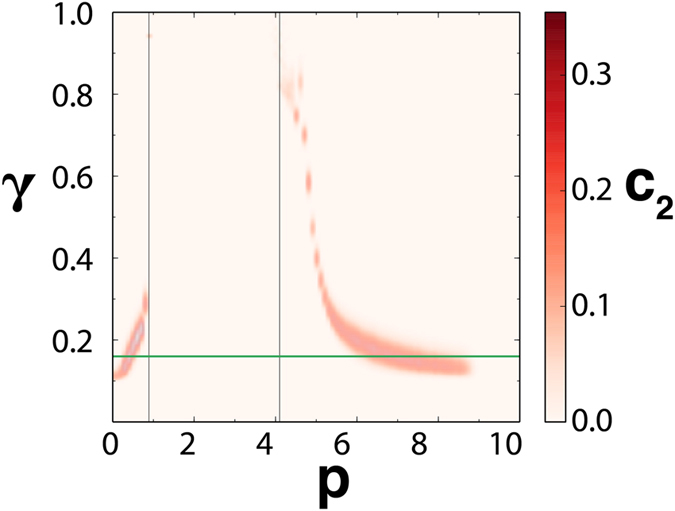
The *c*_2_ values, as given by the color bar, as a function of the control parameter *p* and the threshold *γ* used to build the functional network for the Lorenz’96 system. The dynamical transitions to traveling waves and to chaos are indicated by the vertical black lines. The horizontal green line identifies the value *γ* = 0.16 for which Fig. 5 was constructed.

Functional networks were constructed from *R* = 1000 snapshots by interpreting the locations *k* as nodes and assigning links between pairs of nodes when the Pearson correlation is larger than a threshold *y* = 0.16. Figure 5b shows the quantities *S*_1_, the size of the largest cluster, and 〈*s*^2^〉, the mean cluster size excluding the largest one, for such network. Panels (c,d) display the behavior of *s*_2_, the probability that a randomly chosen node belongs to a cluster of size *s*. These figures clearly identify the presence of a percolated phase at intermediate values of *p*, started and ended by two percolation transitions. Figure 6 shows the quantity *c*_2_ in the (*γ, p*) parameter plane for this model. We see that for increasing threshold the maxima of *c*_2_ approach the locations *p*_1_ and *p*_2_ at which traveling waves are born via a Hopf bifurcation and at which they destabilize into chaotic behavior, respectively. Thus, the percolating phase is a manifestation of the long-range coherence of the traveling wave state, whereas correlation length remains small in the homogeneous and in the chaotic regime. The quantity *c*_2_ (and indeed the other *c*_*s*_) clearly anticipates the first bifurcation when increasing *p*. It also largely anticipates the occurrence of a chaos-order transition when decreasing *p* from large values.

### Percolation in sea temperature networks during El Niño events

To test the behavior of our precursors in observed real situations, we analyze sea surface temperature data from the region of the Pacific used to compute the NINO3.4 index^31^. El Niño-Southern Oscillation^32^,^33^ is the dominant variability mode in present-day climate, characterized by rather irregular (with average period of about 4 years) warm (El Niño) and cold (La Niña) episodes departing from the long-term mean temperature in the equatorial Pacific. These oscillations are related to the presence of a Hopf bifurcation in the coupled atmosphere-ocean system^32^,^33^. The bifurcation can be crossed or just approached, being then the oscillation excited by noise. In both cases there should be a build-up of correlations that would become visible in functional networks constructed from temperature time series. In this case there is no control parameter to fix, but rather the equatorial Pacific evolves in time, coupled to the seasonal cycle, leading to changing spatial correlations. We will see that, despite this lack of control, and without using any information on the underlying dynamics, our approach is able to find precursors of the relevant El Niño-La Niña events.

**Figure 7:**
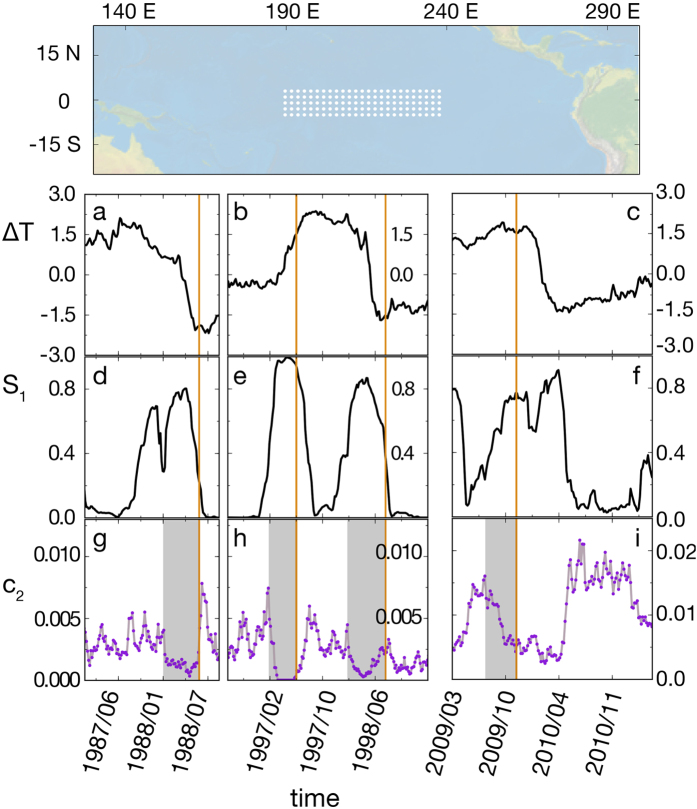
Application to El Niño phenomenon. The upper panel shows a map of the area over which the mean Sea Surface Temperature *T* is monitored in the NINO3.4. Points denote locations used here as nodes in a functional network. The map was generated with Cartopy 0.11.0^36^. Four events, two La Niña (cold) and two El Niño (warm), are shown in the time axis of (panels a–i). Conventional starting dates of the events are marked by vertical orange lines. In (**a**–**c**), the sea surface temperature *T* is shown as a function of time. A functional network is constructed from correlations at *γ* = 0.99992 for 1987–1989 and 1996–1998, and *γ* = 0.9986 for 2009. The size of the giant component *S*_1_ is shown in (**d–f**), showing percolating phases at a plateau, flanked by two percolation transitions, which occurs before each of the events. Panels (g–i) show *c*_2_ in the same time frame. Peaks in *c*_2_ flank both sides of the percolation plateaux, in a manner similar to the Ginzburg-Landau case shown in Fig. 3. The time by which the peaks of *c*_2_ anticipate the conventional starting date of the event is marked in gray.

Sea Surface Temperatures were obtained from the ERA-interim reanalysis of the European Centre for Medium-Range Weather Forecasts^34^, with daily temporal resolution and a spatial resolution of Δ*x* = 0.125°, in the range of years 1979−2014 (Fig. 7). Daily functional networks at day *t* were built from these time series computing the Pearson correlation with a time window of *R* = 200 days (100 days before and 100 days after time *t*). The quantities plotted in Fig. 7 are further averaged over 5 days.

In Fig. 7, we have focused on three different periods: 1987–1989, during which a strong La Niña occurred, 1996–1998, featuring an El Niño-La Niña pair, and a recent El Niño in 2009. In contrast to the previous examples, this system is empirical and the contribution of the noise is more difficult to assess than in a model equation. Therefore, the systematic search for the optimal *γ* has not been performed. Nevertheless, the space of values of *γ* and how they affect *c*_2_ have been explored in Supplementary Figure S1. Interestingly, it seems that the range of values of *γ* necessary to observe peaks in *c*_2_ have moved toward lower *γ* in the early 2000s. We have fixed two values of the threshold to produce Fig. 7: *γ* = 0.99992 for the events of 1987–1989 and 1996–1998, and *γ* = 0.9986 for the event of 2009. In the two cases, this is where a nice compromise between signal-and-noise is found. In a practical situation, the selection of *γ* is not a post-hoc process: one can have a clear idea of the range of values to use from the previous events. Once *γ* is set at a fixed value in this range, if *c*_2_ shows a peak, followed by a sequence of peaks of *c*_3_, *c*_5_, etc., the system is very likely going towards a new El Niño/Niña event. The panels a, b and c of Fig. 7 display the variations of the ocean superficial temperature and also the moments at which an El Niño or La Niña event are officially declared are marked with a vertical orange line. The panels (d–f), on the other hand, depict the time evolution of the size of the largest connected component *S*_1_, which peaks before or on the arrival of the event. *c*_2_, represented in the lower panels (g–i), also shows maxima way before the corresponding peak of *S*_1_. The anticipatory period since the *c*_2_ peaks to El Niño (La Niña) event is marked in gray in every plot. It corresponds to 240 days in 1988 (Fig. 7g), 125 days in 1997 (Fig. 7h), 175 days in 1998 (Fig. 7h) and 115 days in 2009 (Fig. 7i).

## Conclusions

In summary, we have shown that consideration of the percolation transition in functional networks constructed from spatial correlations in extended systems provides powerful anticipatory tools for their dynamical regime shifts. Precursors of the percolation transition itself, such as the probabilities *c*_*s*_ for random nodes to belong to small clusters of size *s*, add extra anticipatory range. This is done by introducing a mesoscopic view of the system, instead of using global or local perspectives as the ones used in previous methods using the system dynamics slowing down, the spatial variability of the order parameter or the clustering and degree distribution of the functional network. Furthermore, the sequence of peaks of *c*_*s*_ provides extra information on the distance still remaining to the (percolation) transition. We note that *γ* and *s* are methodological parameters, so that they can be explored even when far from the dynamical transition. Despite the fact that there exists an optimal *γ* for which the anticipatory power is largest, there is typically a wide range of values of *γ* for which the method works. Furthermore, the sequence of peaks of *c*_*s*_ can give a good hint on the distance to the approaching transition.

The tools presented here work in a variety of transition types, the condition being the increase of spatial correlations when approaching the transition point. This happens generally at least for systems close to bifurcations characterized by critical slowing down and with spatial locations coupled by diffusion. Most of the local bifurcations types satisfy the critical slowing down criterion (see discussion in refs 6,10). Although diffusive coupling is sufficient to provide increasing spatial correlations when combined with critical slowing down, it is by no means necessary, as the example of the Lorenz’96 model (for which spatial coupling is of advection type rather than diffusive) shows.

Through this paper we have focused in spatially embedded complex systems, in which network nodes are associated to spatial locations. Since the only information needed to apply our framework is a set of time series coming from different network nodes, we expect our approach to be also useful in more general network systems experiencing regime transitions^35^, beyond the spatial ones. In addition we have shown that the percolation-based precursors can be used even in cases, such as El Niño events, where very little or no information on the underlying dynamics is available. This is, therefore, a fresh perspective on a known phenomenon with the bonus of offering a method that can become instrumental in the monitoring and management of complex systems.

## Additional Information

**How to cite this article**: Rodríguez-Méndez, V. *et al*. Percolation-based precursors of transitions in extended systems. *Sci. Rep.* **6**, 29552; doi: 10.1038/srep29552 (2016).

## Supplementary Material

Supplementary Information
