# Erratum: Percolation-based precursors of transitions in extended systems

**DOI:** 10.1038/srep30993

**Published:** 2016-08-25

**Authors:** Víctor Rodríguez-Méndez, Víctor M. Eguíluz, Emilio Hernández-García, José J. Ramasco

Scientific Reports 6: Article number: 29552; 10.1038/srep29552published online: 07 14 2016; updated: 08 25 2016

The original HTML version of this Article contained a typographical error in the spelling of the author Víctor M. Eguíluz which was incorrectly given as Víctor M. Eguíluz M. This has now been corrected.

