# Supplementary material for: Percolation-based precursors of transitions in extended systems
Source: Sci Rep. 2016 Jul 14;6:29552. doi: 10.1038/srep29552 (PMC4944164; doi:10.1038/srep29552)
Supplement: Supplementary Information [file srep29552-s1.pdf]

Supplementary Information for Percolation-based precursors of transitions in extended systems

Víctor Rodríguez-Méndez[1], Víctor M. Eguíluz[1], Emilio Hernández-García[1], and José J. Ramasco[1]

[1]Instituto de Física Interdisciplinar y Sistemas Complejos IFISC (CSIC-UIB), 07122 Palma de Mallorca, Spain

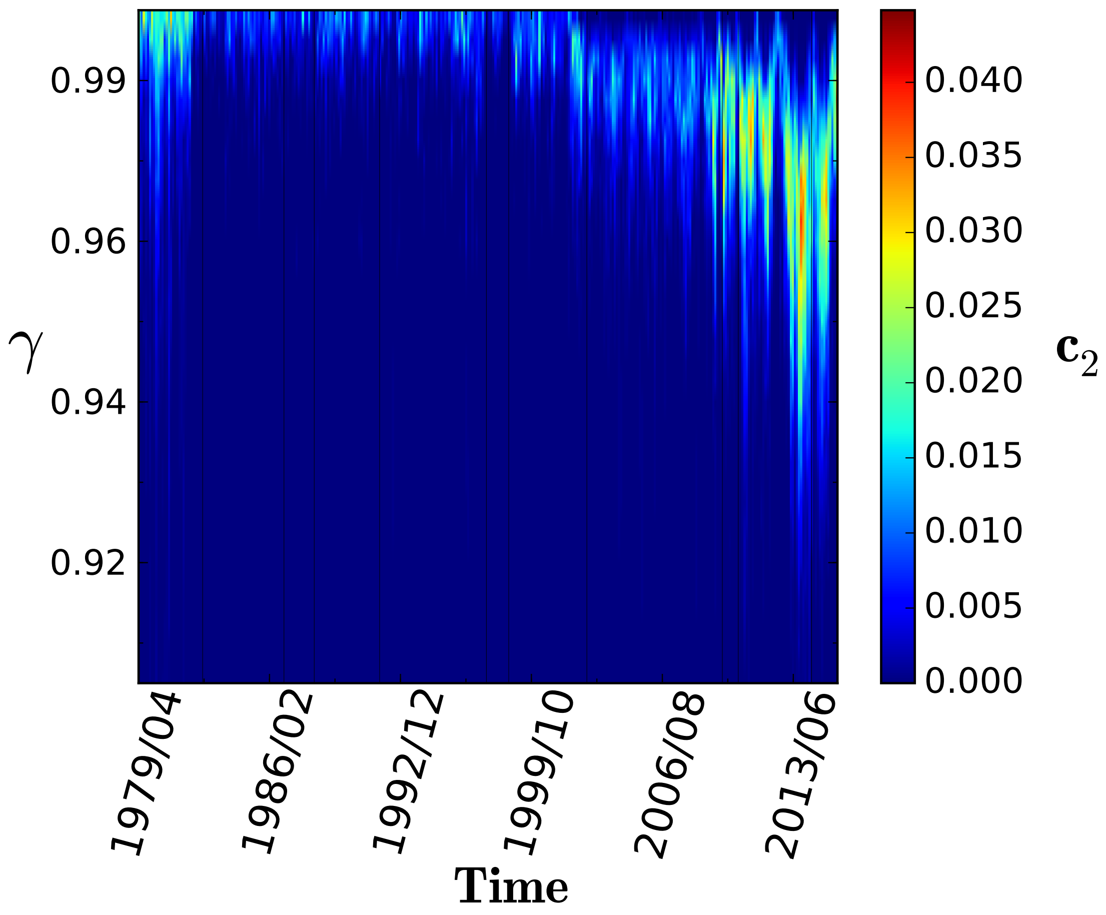

Figure S1: Heatmap with an exploration of the fraction of nodes in clusters of size 2, c_2, as a function of time and of γ for functional networks extracted daily over 30 years of data on the mean Sea Surface Temperature T monitored in the NINO3.4. The spatial resolution is 0.5 arc degrees.
